# Characterization of the chloroplast genome of *Verbena officinalis* Linn. (Verbenaceae) and its phylogenetic analysis

**DOI:** 10.1080/23802359.2021.1914224

**Published:** 2021-05-23

**Authors:** Zhizheng Fang, Guangrong Qian, Nianjun Yu, Rongchun Han, Dezhu Ge

**Affiliations:** a School of Pharmacy, Anhui University of Chinese Medicine, Hefei, China; b Department of Research and Development, Anhui Jiren Pharmaceutical Company, Bozhou, China

**Keywords:** Chloroplast genome, *Verbena officinalis*, phylogenetic analysis

## Abstract

*Verbena officinalis* has a long history as a source plant in traditional Chinese medicine. This study adopted next-generation sequencing technology in order to determine complete chloroplast genome of *V. officinalis*. The results of this investigation showed the chloroplast genome of *V. officinalis* was 153,286 bp in length, including a pair of inverted repeat (IR) regions (each 25,825 bp), separated by a large single-copy region (LSC) of 84,316 bp and a small single-copy region (SSC) of 17,320 bp, and the overall GC contents of the chloroplast genome was 39.04%. Additionally, we annotated 83 genes, including 48 protein-coding genes, 31 tRNA genes, and 4 rRNA genes. By creating the phylogenetic tree, relationship between *V. officinalis* and relevant species was discussed, and the result proved that *V. officinalis* was closely related to *Avicennia marina*. The findings of the study will serve as a stepping stone for follow-up researches regarding its chloroplast genome.

*Verbena officinalis* is a perennial plant species, which has been widely used as a herbal medicine in China for centuries. Besides, both the Chinese Pharmacopeia and the European Pharmacopeia have included *V. officinalis* (Kubica et al. 2020). It is distributed from temperate to tropical regions all over the world. *V. officinalis* grows on roadsides, hillsides, streams or forests at low to high altitudes (Waheed et al. 2016). At present, it is also gradually beginning to be used as an ornamental plant because of its purple flowers. The previous study showed that extracts from the leaves of *V. officinalis* had anti-inflammatory (Deepak and Handa 2000), antioxidant and antifungal activities (Casanova et al. 2008). However, studies on *V. officinalis* were mainly in the fields of population biology, ecology, physiology, and stress resistance. The research on characterization of the chloroplast genome of *Verbena officinalis* is still in infancy, and further investigation is needed.

The genomic DNA of *V. officinalis* was extracted from its freshly-picked leaves which were collected in Heifei, Anhui, China (N31°56′17″; E117°23′25″). The plant specimens were kept in the Herbarium of Anhui University of Chinese Medicine with the voucher number 200809AH001. We took an approach to extract genomic DNA by using CTAB method (Borges et al. 2012). In order to obtain the sequence raw data, we entrusted Genewiz Co. Ltd. (Suzhou, China) to conduct next-generation sequencing. After discarding and trimming reads with low quality, the clean data were assembled and gapfilled by Trinity and SSPACE (version 3.0). Based on the assembly results, the coding gene, rRNA, tRNA and other ncRNAs were predicted, and then the predicted genes were analyzed by functional annotation of KEGG database (Ogata et al. 1999).

The complete chloroplast genome sequence of *V. officinalis* was uploaded to GenBank with the accession number MW348926. The final result showed that genomic sequence length was 153,286 bp, which consisted of a pair of inverted repeat (IR) regions (each 25,825 bp), separated by a large single-copy region (LSC) of 84,316 bp and a small single-copy region (SSC) of 17,320 bp. The GC contents in the chloroplast genome were found to be 39.04%. The chloroplast genome of *Verbena officinalis* contained 83 genes, including 48 protein-coding genes, 31 tRNA genes, and 4 rRNA genes.

The phylogenetic trees help in acquiring the evolutionary history of *Verbena officinalis*. Therefore, to study evolutionary information, we established phylogenetic tree by using MEGA X (Kumar et al. 2018). The analysis was based on complete chloroplast genome sequence of *V. officinalis*, along with other 11 relevant species downloaded from GenBank. In the process, the chloroplast genome sequences were aligned with ClustalW. Subsequently, we chose Bootstrap method to conduct phylogeny test (1000 replicates), with a combining method of Tamura-Nei substitution model (Tamura and Nei 1993) to generate maximum likelihood (ML) tree (Kumar et al. 2016). The finding of this study suggested that *V. officinalis* was closely related to *Avicennia marina* which is from the same family of *Verbenaceae* (Figure 1).

**Figure 1.**
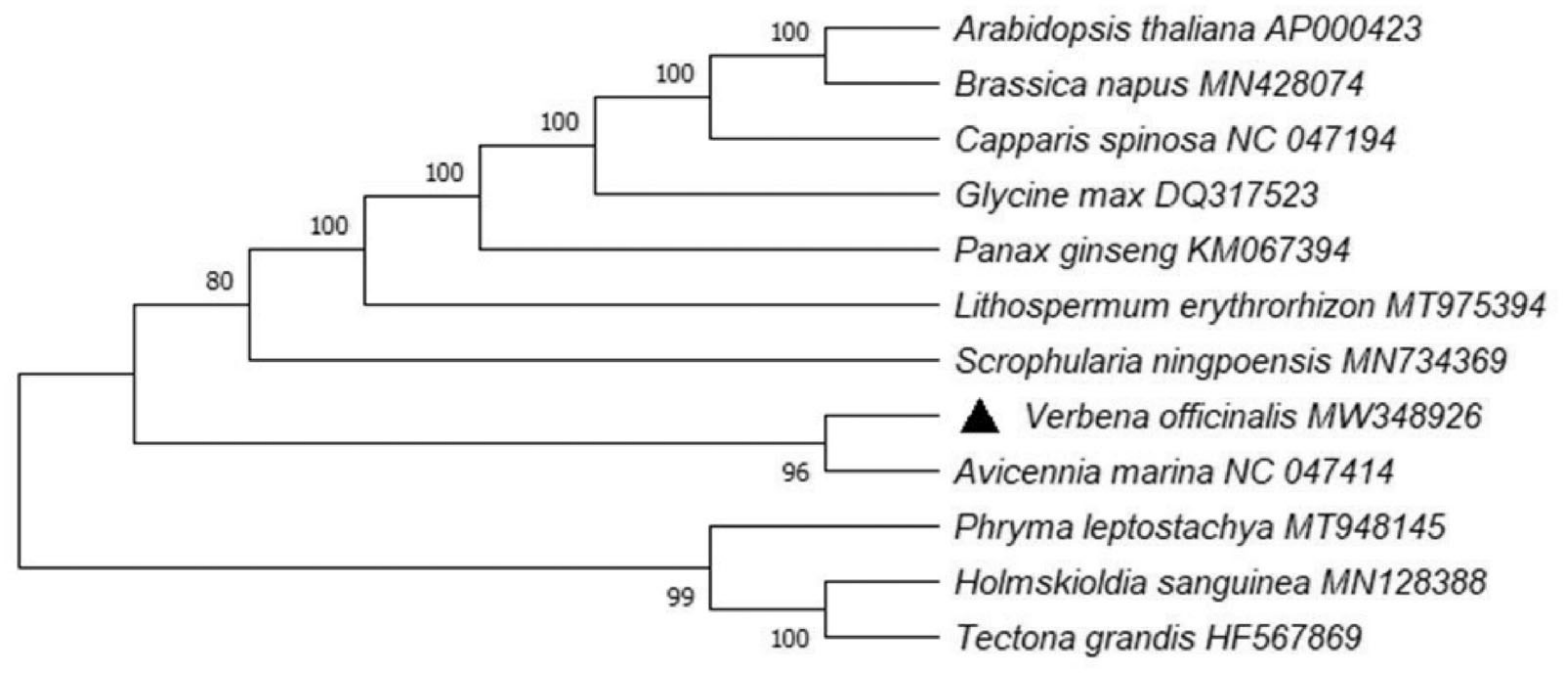
Maximum likelihood phylogenetic tree was constructed by using MEGA X. It was based on complete chloroplast genome sequence of *V. officinalis*, along with selected 11 relevant species downloaded from GenBank.

## Data Availability

The genome sequence data that support the findings of this study are openly available in GenBank of NCBI at https://www.ncbi.nlm.nih.gov under the accession number MW348926. The associated BioProject, Bio-Sample and SRA numbers are PRJNA688015, SAMN17167431, and SRR13309663 respectively.
